# DNA methylation analysis at imprinted locus and global methylation in spermatozoa of normozoospermic individuals of idiopathic Recurrent Spontaneous Miscarriage

**DOI:** 10.1186/1756-8935-6-S1-P2

**Published:** 2013-03-18

**Authors:** Mandar M Ankolkar, Himangi Warke, Vinita Salvi, Neelam K Mokashi, NH Balasinor

**Affiliations:** 1 Department of Neuroendocrinology, National Institute for Research in Reproductive Health, Mumbai, India; 2 Department of Gynecology and Obstetrics, KEM Hospital and G S Medical College, Mumbai, India

## Background

Gametic imprint marks play an essential role in dictating the expression profile to the developing embryo as they are faithfully inherited in the somatic tissues. Insulin-like growth factor(*IGF2*) is a mitogen and *IGF2-H19* locus, a paternally imprinted locus, has been found to be associated with embryo loss in numerous publications. Aberrations in its methylation levels in tamoxifen treated rats evidenced despite any change in sperm parameters [1]. *DLK1*(Drosophila-like homologue1) is also involved in embryogenesis and the *IGDMR*(a paternally methylated locus) methylation aberrations has been found to be associated in oligozoospermic patients [2]. Recurrent Spontaneous Miscarriage (RSM) is the spontaneous consecutive loss of >3 pregnancies in the first trimester. Although many factors have been attributed to its cause many cases remain idiopathic and undiagnosed. Based on evidences supporting the prevalence of paternal epigenetic marks associating with embryo loss we hypothesized the involvement of aberrant methylation of imprinted genes *IGF2-H19* imprint control region, *DLK1-GTL2* IGDMR and global methylation in spermatozoa of idiopathic Recurrent Spontaneous Miscarriage (RSM) case participants having normozoospermic profile in order to understand its involvement in causing early embryo loss in these cases.

## Materials and methods

Spermatozoa of 52 normozoospermic male participants (26 Control and 26 RSM) were analyzed for concentration, motility, morphology and chromatin compaction. Genomic DNA was extracted, treated with bisulfite reagent and cloned in a subcloning vector. 10-15 clones were sequenced and analyzed for methylation of the *H19 ICR* and *DLK1-GTL2 IGDMR* and the methylation was quantified. Global methylation was analyzed using the anti-5-methyl cytosine antibody.

## Results

We observed significant hypomethylation in the *H19 ICR* but not in global methylation and the *IGDMR* locus. Hypomethylation of the *H19 ICR* was significant at p<0.01 [3]. Hypomethylation at *H19 ICR* was observed in terms total CpG methylated in the locus and the CTCF6 binding region, and the no. of completely methylated clones. Sperm parameters analyzed did not differ significantly in the control versus the RSM group for neither of the parameters analyzed, thus supporting our hypothesis further.

## Conclusion

Our results indicate the prevalence of DNA methylation aberration at the *H19 ICR* locus in idiopathic RSM despite normozoospermic semen profile. It opens up further avenues of research in identifying more such epigenetic markers and to understand the prevalence and effect of paternal DNA hypomethylation of imprinted genes in causing embryo loss in such cases.
